# A chronic bioluminescent model of experimental visceral leishmaniasis for accelerating drug discovery

**DOI:** 10.1371/journal.pntd.0007133

**Published:** 2019-02-14

**Authors:** Raquel Álvarez-Velilla, Maria del Camino Gutiérrez-Corbo, Carmen Punzón, Maria Yolanda Pérez-Pertejo, Rafael Balaña-Fouce, Manuel Fresno, Rosa María Reguera

**Affiliations:** 1 Departamento de Biología Molecular, Centro de Biología Molecular Severo Ochoa, Consejo Superior de Investigaciones Científicas, Universidad Autónoma de Madrid, Madrid, Spain; 2 Departamento de Ciencias Biomédicas, Universidad de León, León, Spain; 3 Diomune S.L Parque Científico de Madrid, Madrid, Spain; University of Antwerp, BELGIUM

## Abstract

**Background:**

Visceral leishmaniasis is a neglected parasitic disease with no vaccine available and its pharmacological treatment is reduced to a limited number of unsafe drugs. The scarce readiness of new antileishmanial drugs is even more alarming when relapses appear or the occurrence of hard-to-treat resistant strains is detected. In addition, there is a gap between the initial and late stages of drug development, which greatly delays the selection of leads for subsequent studies.

**Methodology/Principal findings:**

In order to address these issues, we have generated a red-shifted luminescent *Leishmania infantum* strain that enables long-term monitoring of parasite burden in individual animals with an *in vivo* limit of detection of 10^6^ intracellular amastigotes 48 h postinfection. For this purpose, we have injected intravenously different infective doses (10^4^—5x10^8^) of metacyclic parasites in susceptible mouse models and the disease was monitored from initial times to 21 weeks postinfection. The emission of light from the target organs demonstrated the sequential parasite colonization of liver, spleen and bone marrow. When miltefosine was used as proof-of-concept, spleen weight parasite burden and bioluminescence values decreased significantly.

**Conclusions:**

*In vivo* bioimaging using a red-shifted modified *Leishmania infantum* strain allows the appraisal of acute and chronic stage of infection, being a powerful tool for accelerating drug development against visceral leishmaniasis during both stages and helping to bridge the gap between early discovery process and subsequent drug development.

## Author summary

Visceral leishmaniasis is a neglected disease that poses a significant threat to impoverished human populations of low-income countries. Due to the unavailability of vaccines, pharmacological treatment is the only approach to control the disease that otherwise can be lethal. To date, drug management in endemic regions is based on combinations of a handful of mostly unsafe drugs, where the emergence of resistant strains is an additional problem. To accelerate the discovery of new drug entities, several gaps from the early discovery of a compound to its public use, should be filled. One of these gaps is the need of a rapid go/no-go testing system for compounds based on robust preclinical models. Here, we propose a new long-term model of murine visceral leishmaniasis using *in vivo* bioluminescent imaging. For this purpose, a red-shifted bioluminescent *Leishmania infantum* strain was engineered. This strain has allowed the appraisal of the disease in individual animals and the monitoring of parasite colonization in liver, spleen and bone marrow. As proof of concept of this platform, mice were infected with the transgenic *L*. *infantum* strain treated with a standard schedule of miltefosine, the only oral drug available against *Leishmania* parasites. Bioluminescence and parasite load in the target organs were compared showing a good correlation. Our findings provide a robust and reproducible tool for drug discovery in a chronic model of murine visceral leishmaniasis.

## Introduction

Leishmaniasis is a complex of neglected parasitic diseases affecting the poorest people in 98 countries, particularly those with weak or non-existent health systems. [1]. There are at least three different forms of clinical presentations; cutaneous, mucocutaneous and visceral leishmaniasis, the latter being fatal if left untreated [2]. Visceral leishmaniasis (VL) is estimated to produce 300.000 new cases and between 20.000–40.000 deaths every year. Most of the cases are localized in three geographical regions; South Asia and East Africa where the disease is caused by *Leishmania donovani* and the transmission is mostly anthroponotic. By its part, in Brazil, where the disease is produced by *L*. *infantum chagasi*, the transmission is zoonotic and occurs mainly from infected dogs [3].

Nowadays, therapeutic or prophylactic human vaccines are still lacking, and the cure of patients is based on chemotherapy [4, 5]. Treatment of VL was mainly based on painful intramuscular injections of pentavalent antimonials, such as sodium stibogluconate (SSG). SSG has been the first-line antileishmanial drug in India, although its clinical efficacy in some areas of North Bihar State has gradually declined, due to the emergence of fully resistant *L*. *donovani* strains. SSG is being substituted by liposomal amphotericin B (AmBisome) as first-line treatment, despite slow intravenous administration of the drug is needed [6–8]. In East Africa, SSG was the first-line regimen for decades, but due to its toxicity and following WHO recommendations in 2010, SSG + paromomycin combination therapy became the treatment of choice [9]. However, the administration of this drug combination is painful and requires patient hospitalization, and therefore, more friendly alternatives were implemented. These include single dose of AmBisome plus 10 consecutive days of SSG, single dose of AmBisome plus 10 days of miltefosine or miltefosine alone for 28 days. However, none of these combinations improved the results of the treatment of choice in Phase II clinical trials [10].

Miltefosine is the last drug successfully introduced against VL. It is also the only drug that has a good oral bioavailability. However, an increase in relapse rates has been reported in India and Nepal, probably associated with low drug exposure [11, 12]. In addition, miltefosine is potentially embryotoxic and fetotoxic in experimental animals and thereby, its administration is not recommended in women during pregnancy [13].

For all these reasons, there is an unmet need to fill the antileishmanial drug discovery pipeline with safer drugs that display new mechanisms of action, likely allowing combination therapy in order to prevent the emergence of resistant strains [14]. During this process, and once compounds have shown high *in vitro* potency, selectivity, specificity, low toxicity and good predictable pharmacokinetic/pharmacodynamic properties, a proof of concept that undoubtedly shows the *in vivo* efficacy of lead compounds, is required. Both mice and hamsters are used as models of acute and chronic VL, respectively, during the evaluation of the proof of concept [15, 16]. The most frequent technique to evaluate the infection course after drug treatment has been microscopic counting of amastigotes in liver, spleen and bone marrow smears stained with Giemsa dye. However, these are labour-intensive techniques that require specific skill training and present low sensitivity when parasite burdens are low after treatment [17], therefore, they are limited by tissue sampling biases, which require large animal cohorts.

*In vivo* real-time imaging combined with modified parasites expressing bioluminescent or fluorescent reporters may accelerate the initial stage of drug discovery at the preclinical level. Fluorescent reporters in the near infrared wavelength avoid interference with haemoglobin and do not require the addition of substrate. However, and despite the number of near infrared proteins currently available [18, 19] further reporters with longer emission wavelengths are still required in order to increase sensitivity. In this regard, red-shifted bioluminescent reporters [20] are currently allowing the appraisal of different infections produced by Trypanosomatids *in vivo* in real-time without the need to kill animals. This tool allows to run longitudinal studies with a reduced number of animals since they are not sacrificed, and in addition each animal is its own control, therefore the variability of experimental outcomes is limited [21–25]. In summary, *in vivo* real-time imaging allows to develop the proof of concept in a record time, accelerating the drug discovery process.

Nowadays, the mouse is used as acute preclinical model of VL, being liver the main affected organ when experimental treatments are initiated at early times postinfection. On the contrary, hamster is a more stringent and relevant model to recreate human VL [26, 27]. Generally speaking, during chronic infections the persistence of pathogens yields a state of T cell dysfunction known as exhaustion that is characterized by the loss of effector functions, low recall response and suboptimal T cell proliferation [28]. This is a hallmark feature shared by mice, dogs and humans and it is associated with disease progression [29–31].

Here, we describe a chronic murine model of VL that combines *in vivo* real-time image with stably modified strain expressing red-shifted luciferase (luc) aiming to track the presence of parasites in target organs during a long-time course of infection that can be used for preclinical drug-discovery. Miltefosine was used as proof of concept to assess the suitability of this technique during drug discovery.

## Methods

### Ethics statement

The animal research described in this manuscript complies with Spanish Act (RD 53/2013) and European Union Legislation (2010/63/UE). The protocols were approved by the Animal Care Committee of the Centro de Biología Molecular Severo Ochoa (CBMSO, Madrid, Spain), project licence number JMJ/bb. Animals were maintained under specific pathogen-free conditions in individually ventilated cages. They experienced a 12 h light/dark cycle and had access to food and water *ad libitum*.

### Methods, mice and parasites

Seven to eight weeks-old female Balb/c mice were obtained from Janvier Labs (St Berthevin Cedex, France) and housed in specific pathogen-free facilities in the P2-facility of CBMSO for this study. *L*. *infantum* (strain MCAN/ES/96/BCN 150) promastigotes (previously obtained from infected dogs) were a gift from J.M. Requena (CBMSO, Madrid, Spain). Parasites were routinely cultured at 26 °C in M199 medium supplemented with 25 mM HEPES pH 6.9, 10 mM glutamine, 7.6 mM hemin, 0.1 mM adenosine, 0.01 mM folic acid, 1x RPMI 1640 vitamin mix (Sigma-Aldrich), 10% (v/v) heat-inactivated fetal calf serum (FCS) and antibiotic cocktail (50 U/ml penicillin, 50 μg/ml streptomycin).

### Generation of red-shifted luc *L*. *infantum* strain

The 1647-bp *PpyRE9h* coding region was amplified by PCR from pGEX-6P-2 HCO RE9h vector, a kind gift from Dr. Bruce Branchini, (Department of Chemistry, Connecticut College, CT, USA). The oligonucleotides used as primers (RBF919 and RBF920 in Table 1) introduced *Nco*I-*Not*I as restriction sites for cloning into pLEXSY-PAC vector (Jena Bioscience) and *Xho*I restriction site in the forward primer for cloning into pSK II vector. The 1647-bp PCR amplified fragment containing the PpyRE9h coding region was digested with *Xho*I and *Not*I and cloned first into pSK II vector previously cut with the same restriction enzymes to yield pSK-PpyRE9h plasmid. Then, this plasmid was cut with *Nco*I-*Not*I and the *PpyRE9h* ORF was cloned into pLEXSY-PAC vector to yield the pLEXSY-PAC-PpyRE9h construct. Parasites expressing red-shifted luc were obtained after electroporation of *L*. *infantum* BCN150 promastigotes with the linear *Swa*I-targeting fragment obtained from pLEXSY-PAC-PpyRE9h vector. Transfections were performed by electroporation (Gene Pulser X cell System, Biorad) using 10 μg of DNA fragments under the following conditions: 25 μF, 1500 v, 9 ms in 4 mm gap cuvettes. Subsequent plating on semisolid media containing 200 μg/mL puromycin as selection antibiotic, allowed the isolation of individual colonies that were subcultured in liquid media under antibiotic pressure. The correct integration of each fragment into the 18S rRNA locus of the resulting clones (*PpyRE9h+L*. *infantum*) was confirmed by PCR amplification analysis, using appropriate primers (Table 1).

**Table 1:** Oligonucleotides used in this work.

Oligo No.	Sequence^a^^,^^b^	Purposed^c^	Orientation
RBF919	ccgCTCGAG*CCATGG***CCACC**ATGGAGGACGCCAAGAACATCAA	PpyRE9h	Forward
RBF920	aaggaaaaaa*GCGGCCGC*TCAGATCTTGCCGCCCTTCTTGG	PpyRE9h	Reverse
RBF 630	CTTGTTTCAAGGACTTAGCCATG	5′integration	Forward
RBF 637	TATTCGTTGTCAGATGGCGCAC	5′integration	Reverse
RBF 644	CATGTGCAGCTCCTCCCTTTC	3′integration	Forward
RBF 645	CCTTGTTACGACTTTTGCTTC	3′integration	Reverse

^a^Underlined sequence indicates restriction site.

^b^Bold sequence indicates optimized translation initiation sequence.

^c^Orientation of primers: F, forward; R, reverse

### *In vitro* luciferase assay and microscopy

The Luciferase Assay System (Promega) was used to assess luc expression in PAC-isolated clones from semisolid *in vitro* cultures. Briefly, 100×10^6^ parasites were washed off with PBS and then lysed with 1 ml of Cell Culture Lysis Reagent provided by the manufacturer. Cell lysate was serially diluted in 96-well plate and then, ten microlitres of each cell lysate dilution were mixed with 90 μL of luciferin substrate. Luminescence was measured immediately using a Synergy HT microplate reader (BioTek).

In order to recover the infectivity of *PpyRE9h+L*. *infantum* strain after cloning, 10^8^ metacyclic promastigotes were inoculated intravenously (IV) in the tail vein. Mice were sacrificed 4 weeks later and spleens were used to recover infective amastigotes. Amastigotes were isolated from the spleen by passing the tissue through a wire mesh. Then, splenocytes were disrupted by passing sequentially through 27G1/2 and 30G1/2 needles. Finally, cell debris was retained by passing successively through polycarbonate membrane filters with pore sizes of 8 μm, 5 μm and 3 μm (Isopore, Millipore). Released amastigotes (free of host cells), were washed twice with PBS (4000 x g for 20 min at 4°C) and counted by direct microscopy. To assess luciferase expression in intracellular infections, phorbol 12-myristate 13-acetate (PMA)-differentiated THP1 human monocytic leukemia cells, were grown on 8-well chambered coverslips (IBIDI) and incubated with infective amastigotes recuperated from mice in a ratio of 1:10 for further 4 hours. Extracellular parasites were removed by extensive washing with warm PBS and processed for immunofluorescence analysis 5 days after infection. Briefly, coverslips were fixed with 2% (v/v) paraformaldehyde in PBS and incubated with 0.1% (v/v) Triton X-100 in PBS for 10 min at room temperature in order to permeabilize the cells. Slides were then probed with 1:1000 red firefly luciferase polyclonal antibody (ThermoFisher); followed by 1:500 DyLight 633-conjugated goat anti-rabbit IgG secondary antibody for 30 min at room temperature. DNA was labelled using 1 μM Hoechst 33342 before mounting with Vectashield mounting medium. Images were acquired on a Zeiss LSM800 microscope with airyscan at 60X magnification.

### *In vivo* mice infection with *PpyRE9h+L*. *infantum* parasites. Bio-luminescent imaging (BLI)

Different infective doses of stationary phase *PpyREh9+L*. *infantum* promastigotes (ranging from 5x10^4^ to 5x10^8^) were IV injected to 6–8 weeks-old female Balb/c mice. Every week animals were placed in a Charge-Coupled Device (CCD) IVIS 100 Xenogen system (Caliper Life Science) for BLI analysis and images were acquired 10–20 min after intraperitoneal D-luciferin injection (150 mg/kg). Briefly, the animals were lightly anesthetized with 2.5% isofluorane (then reduced to 1.5%), before being placed on the camera. To standardize image capture and in order to allow comparison between mice, the images presented in the figures correspond to an acquisition time of 1 min duration, taken once luminescence plateaued.

To estimate the parasite burden in living mice, Regions Of Interest (ROIs) around liver and spleen in ventral and lateral animals’ positions were drawn using Living Image v.4.3 to quantify BLI expressed as radiance (p/s/cm^2^/sr). The detection threshold for *in vivo* imaging was estimated using uninfected mice placed in different positions (ventral and lateral), using ROIs of whole animals (n = 16).

At the end of some experiments, animals were euthanized and dissected, to confirm parasite burden by more conventional methods. Briefly, spleens and livers were reimaged *ex vivo* and used to quantify parasite load by Limit Dilution Assay (LDA). LDA was calculated as the geometric mean of the titer obtained from quadruplicate cultures x reciprocal fraction of the homogenized organ added to the first well. The titer was the reciprocal value of the last dilution in which parasites were observed [32].

### Statistic analysis

Individual animal values were used as the unit of analysis of *in vivo* and *ex vivo* experiments. Statistical differences between groups were evaluated using t-student test using SigmaPlot v.14.0. Differences of P < 0.05 were considered significant.

## Results

### Highly sensitive *in vitro* imaging of *L infantum* expressing red-shifted luc

**Fig 1:**
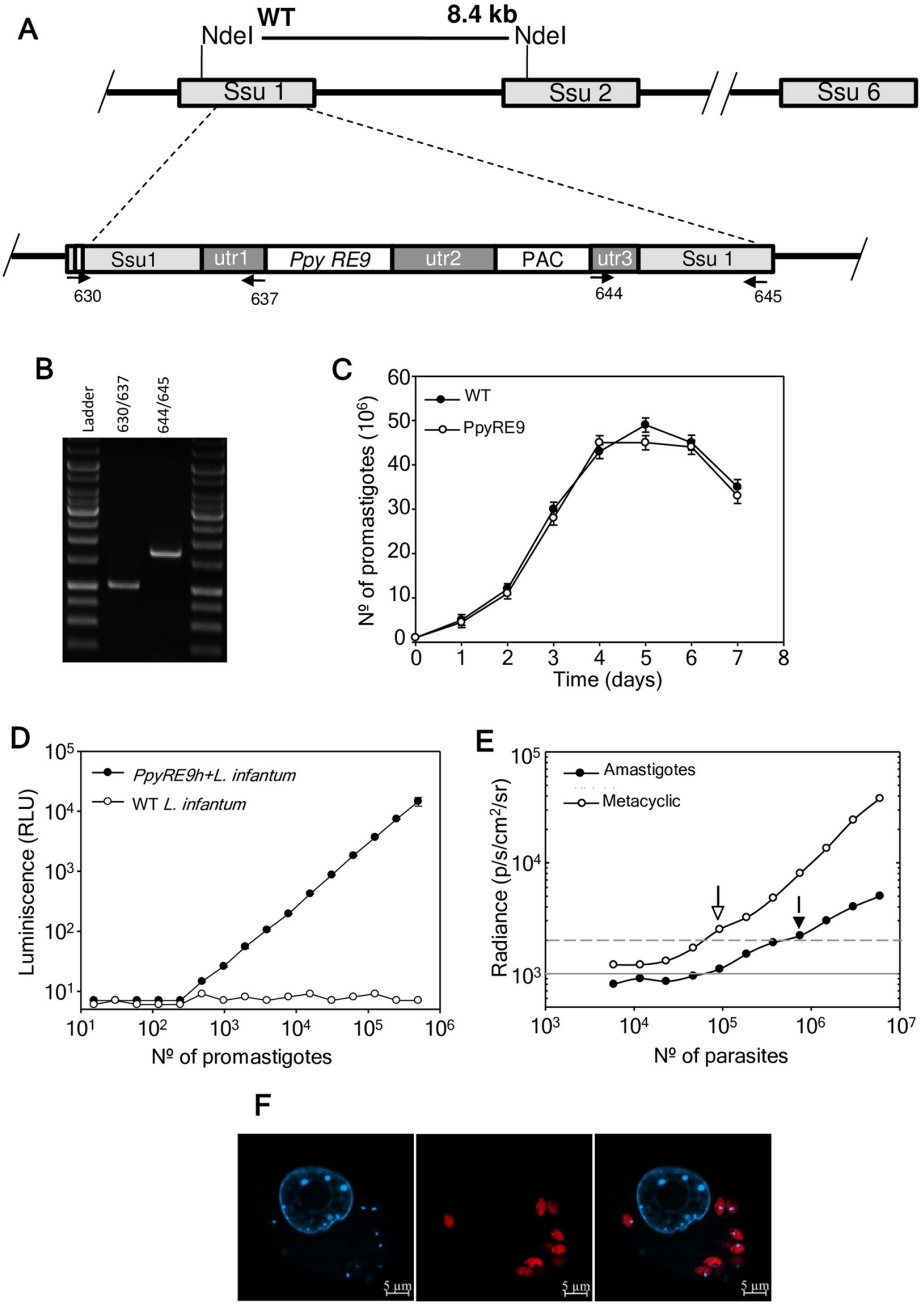
Generation of a *L*. *infantum* stably expressing PpyRE9h red-shifted luciferase. A) Scheme of the structure of the 18S rRNA locus on wild type and planned integration of *PpyRE9h* gene. Key: utr1: non-translated region of *aprt* gene; utr2: 1.4 kb intergenic region from cam operon; and utr3: UTR of *dhfr-ts* gene; PAC; puromycin resistance cassette. B) PCR confirmation of successful integration of the reporter cassette. Primers 630/637 and 644/645 together confirm the correct integration of the reporter cassette into genome sequences. (see Table 1 for sequences). C) Growth rate of wild-type (black circle) and stably-modified promastigotes *PpyRE9h*+*L*.*infantum* (white circle). Parasites were counted using a Coulter counter. D) *In vitro* luciferase activity assay of diluted lysates from wild-type (WT) and *PpyRE9h+L*.*infantum* promastigotes after PCR confirmation. E) Minimal metacyclic promastigote and infective amastigote number detectable by the IVIS camera. Parasites were loaded in 96-well plates, D-luciferin added and the BLI signal was detected in the IVIS camera. Grey lines indicate detection thresholds determined as the mean (solid line) and mean +2SDs (dashed line) of background luminescence of wells with PBS free-parasites. F) Microscopic image of intracellular *PpyRE9h+L*.*infantum* amastigotes infecting PMA-differentiated THP-1 cell line. Bioluminescent amastigotes stained with anti-luciferase IgG (αLuc, red) and Hoechst 33342 DNA stain (H33342, cyan).

*PpyRE9h* was stably integrated into the 18S rRNA promoter using pLEXSY vector (Fig 1A). Correct integration of reporter gene into the resulting clones (*PpyRE9h+L*. *infantum*) was confirmed by PCR amplification analyses using the primers of Table 1 (Fig 1B). The PCR-confirmed clones were screened for luciferase activity and those having higher activity were selected for *in vivo* experiments. Promastigote cultures of *PpyRE9h+L*.*infantum* grew at the same rate as wild-type parasites (Fig 1C). There was a linear relationship between the luciferase activity *in vitro* and the number of parasites independently of the parasite stage (logaritmic, metacyclic or freshly isolated amastigotes) and independently of the instrument for measuring (luminometer or IVIS camera). Fig 1D shows the relationship between luciferase activity and the number of logaritmic promastigotes (*PpyRE9h+L*.*infantum* and wild-type strains). Cell lysates from promastigotes were serially diluted into 96-well plate, D-luciferin was added and luciferase activity was measured using a luminometer (10^3^–10^6^ cells range, r^2^ = 0.999), being the detection limit of 10^3^ promastigotes/well. The PCR-clone with the highest luciferase activity was selected for recovering infectivity through mouse (see Materials and Methods). Fig 1E shows the outcomes using metacyclic promastigotes and free amastigotes in the IVIS camera. In this experiment 6x10^6^ parasites (metacyclic and amastigotes) were serially diluted in a 96-well plate, 100 μl D-luciferin were added and luciferase activity was measured in the IVIS camera (4.68x10^4^-6x10^6^ cells range, r^2^ = 0.987 for metacyclic and r^2^ = 0.994 for amastigotes). The detection limit was 9.37x10^4^ metacyclic/well and 7.5x10^5^ amastigotes/well. This suggests that more amastigotes than metacyclic promastigotes are required for their detection by the IVIS camera.

Spleen-isolated amastigotes were used to infect PMA differentiated THP-1 macrophages. Four hours later the non-phagocytosed parasites were gently washed off with warm PBS and left for further 96 h. The infection was stained using anti-firefly luciferase antibody and its localization was confirmed to be cytosolic by confocal microscopy (Fig 1F).

### Threshold sensitivity of bioluminescence *PpyRE9h+L*. *infantum* strain *in vivo*

**Fig 2:**
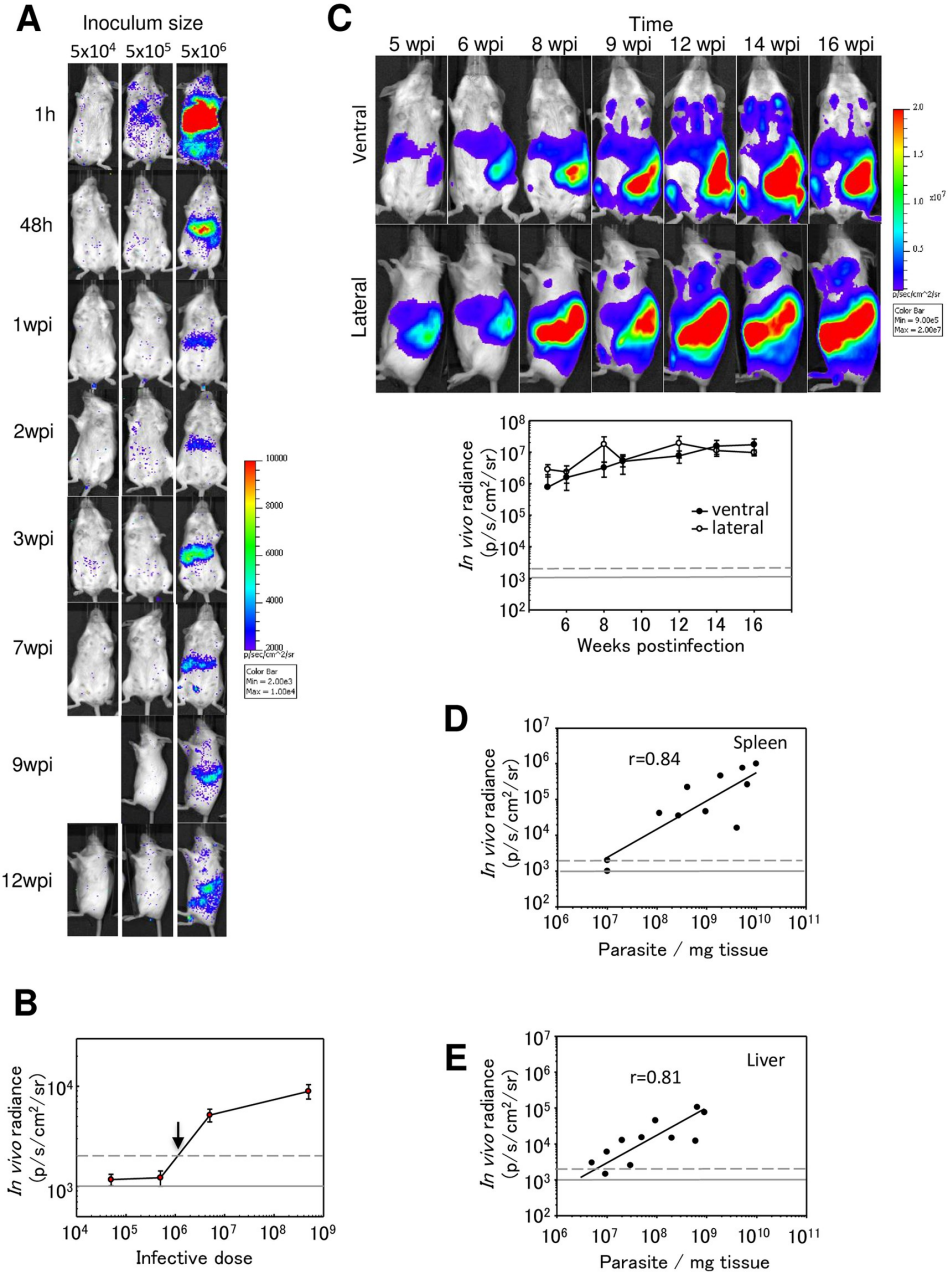
Evaluation of *L*. *infantum* infection in Balb/c mice by *in vivo* BLI. A) Representative images of Balb/c mice infected via IV with 5x10^4^—5x10^6^ metacyclic *PpyRE9h* luciferase-expressing promastigotes. Pseudocolour heat-maps indicate intensity of bioluminescence from low (blue) to high (red). All images use the same log10 scale heat-map, minimum and maximum radiance values are indicated. Animals at 9 and 12 weeks postinfection are in lateral position where most of the BLI signal was detected B) Quantification of whole animal total bioluminescence for mice in the experiment illustrated in A) at 48 h postinfection. C) Time-appraisal of Balb/c mice after IV injection with 5x10^8^ red-shifted bioluminescent parasites (*PpyRE9h+L*.*infantum*) in ventral and lateral positions. Quantification of ventral and lateral bioluminescence from the experiment represented by the images in C). D-E) Correlation between *in vivo* bioluminescence values and parasite burdens in the liver and the spleen. Bioluminescence was measured *in vivo* in ROIs around liver and spleen and parasite numbers were quantified by limiting dilution assay after animals were sacrificed. Grey lines indicate detection thresholds determined as the mean (solid line) and mean +2SDs (dashed line) of background luminescence of control uninfected animals.

The infectivity of the selected clone was enhanced by passing through Balb/c mice that were successively infected with 10^8^ promastigotes by IV route until spleen weight increased up to 0.7–1 g (4–5 passages through mice). To establish the *in vivo* sensitivity of the *PpyRE9h*+*L*. *infantum* strain, Balb/c mice were IV injected with different doses of infective metacyclic promastigotes (5x10^4^-5x10^6^) and photographed 1 h postinfection. At this time, the bioluminescent signal was detected in the liver but only with the highest doses (5x10^5^ and 5x10^6^). Forty-eight hours later when most promastigotes have transformed into amastigotes; BLI signal was only detected from mice infected with 5x10^6^ parasites (Fig 2A). The appraisal of the infection showed that BLI signal in the liver peaked ~3 weeks post-infection (acute phase), then disappeared slowly from this organ and increased in the spleen (chronic phase) (Fig 2A). To estimate the *in vivo* limit of detection with *PpyRE9h+L*. *infantum* parasites, we used BLI signal expressed as radiance (p/s/cm^2^/sr). The limit of detection *in vivo* was estimated to be above 1x10^6^ parasites at 48 h postinfection, when metacyclic parasites have transformed into intracellular amastigotes (Fig 2B).

We were interested in developing a chronic model of infection to use as proof of concept for well-established infections in spleen and bone marrow. In order to evaluate the stability of bioluminescent signal through time in chronic infections, 5x10^8^ metacyclic promastigotes were IV injected and animals were photographed in ventral and lateral positions from 5 to 16 weeks post-infection. The spleen infection was detected independently of the animal positions. BLI signal was increasing from week 5 to reach the maximum radiance 12 weeks after infection (Fig 2C). Moreover, bone marrow radiance was detected only in ventral images from 8 to 16 weeks, and the bioluminescent signal was increasing during this time (Fig 2C).

To establish a correlation between BLI signal detected *in vivo* and the parasite burden in liver and spleen, mice (n = 12, one animal died before the end of the experiment) were infected with parasite dose ranging from 5x10^6^-5x10^8^. Nine weeks postinfection, animals were imaged and the luminescence was recorded *in vivo* in the regions of interest (ROI) previously drawn around the spleen and liver. Animals were euthanized and the liver and spleen processed to determine parasite burden. Both organs showed a good correlation with the *in vivo* recorded BLI signal (Fig 2D and 2E).

### Validation of the luciferase model with miltefosine

*PpyRE9h+L*. *infantum* infected mice were treated with miltefosine as a proof-of-concept to validate this model in a long-term follow-up infection.

**Fig 3:**
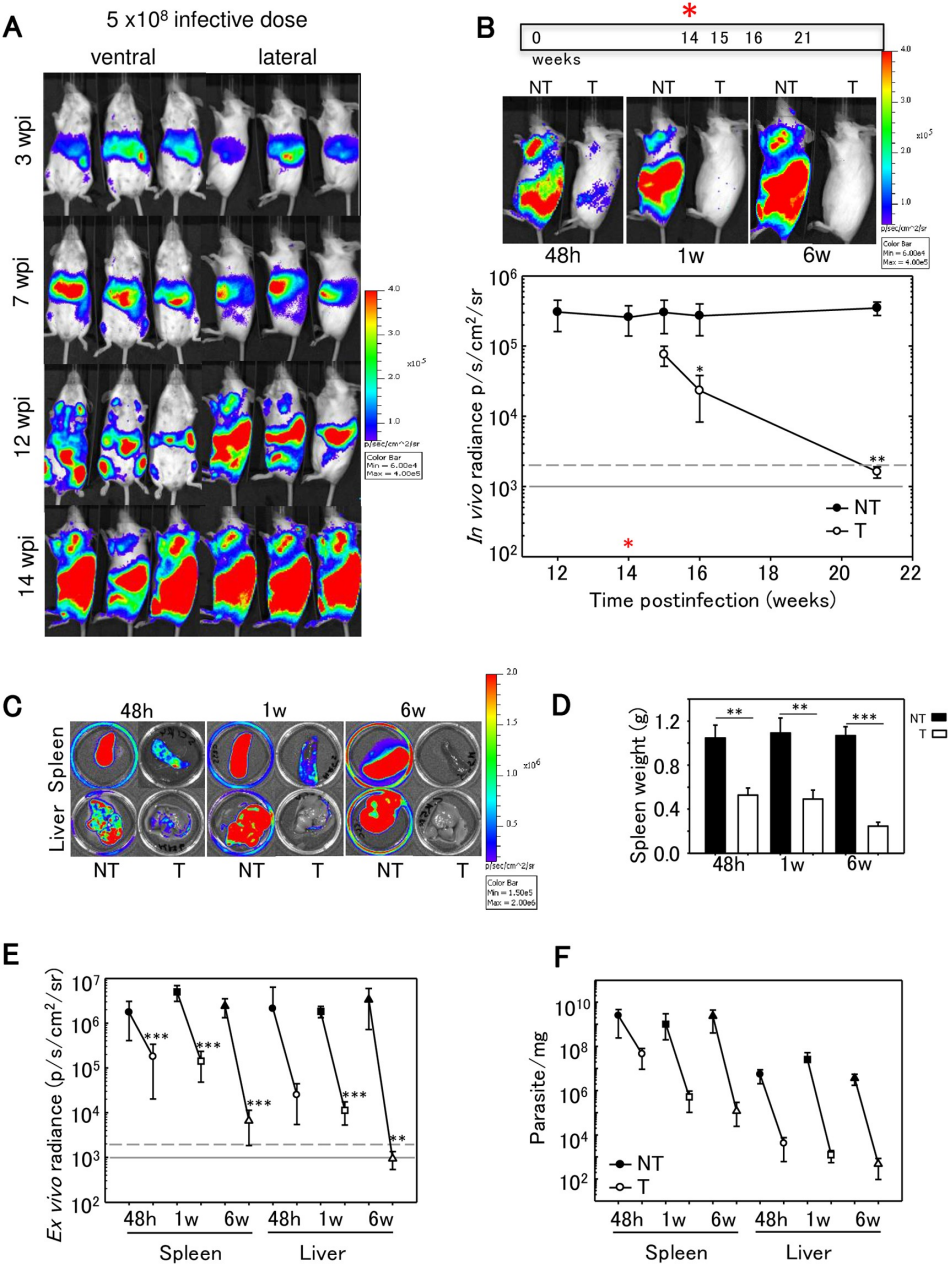
Chronic *L*. *infantum* infection in space and time. A) Representative ventral and lateral view images of Balb/c mice taken at sequential time points over the course of 14 weeks after IV infection with 5 x 10^8^ *PpyRE9h* luciferase-expressing *L*.*infantum* metacyclic promastigotes (representative of n = 30). In the images corresponding to 14 wpi only lateral views are shown because most of BLI signal was detected in this position. Heat-maps are on log10 scales indicate intensity of bioluminescence from low (blue) to high (red); the minimum and maximum radiances for the pseudocolour scale are indicated. B) Animals (n = 15) were treated with 40 mg/kg/d miltefosine via oral for 5 days. Treated and untreated animals were photographed, sacrificed and the spleen and liver imaged at 48 h, 1 week and 6 weeks after the end of miltefosine treatment (15, 16 and 21 wpi). Quantification of lateral bioluminescence for mice shown in B. The red asterisk indicates the start of miltefosine treatment. *In vivo* radiance from untreated (black circle) and treated (white circle) animals is represented. Black asterisks indicate P-values for t-student test (B,D,E). Comparisons between miltefosine treated groups and untreated control groups (*P < 0.05; **P<0.01; ***P<0.001). Grey line indicates detection thresholds determined as the mean (solid line) and mean +2SDs (dashed line) of background luminescence of control uninfected animals. C) *Ex vivo* imaging (spleen and liver) in untreated and treated animals at 48h, 1w and 6 w after the end of the treatment (BLI signal results from the D-luciferin injected *ex vivo*). D) Spleen weights from untreated (black) and treated (white) animals at 48h, 1w and 6 w after the end of the treatment. Each point is the mean ± SD, n = 5 per group. E) *Ex vivo* bioluminescence signal from spleens and livers obtained from untreated (black symbols) and treated animals (white symbols) at 48 h (circle), 1w (square) and 6w (triangle) after the end of miltefosine treatment. F) Parasite burdens in untreated and treated mice determined by limited dilution assay on livers and spleens.

Mice (n = 30) were IV infected with 5x10^8^ metacyclic promastigotes and imaged for BLI after 3, 7, 12 and 14 wpi confirming that infection was established (Fig 3A). Animals were divided in groups and half of them were treated with miltefosine 40 mg/kg/day for 5 days by oral gavage. Once miltefosine treatment was ended, animals were imaged and sacrificed at different times (48 h, 1 and 6 weeks post-treatment that corresponded to 15, 16 and 21 wpi). Fig 3B (top panel) shows that BLI signal in whole animals was almost undetectable after miltefosine treatment (48 h post-treatment) in a chronic infection, and that the BLI reduction persisted for 6 weeks after the end of the treatment. Quantification of the BLI signal revealed that radiance in untreated animals (3x10^5^ p/s/cm^2^/sr) decreased significantly to 2.32x10^4^ and 1.61x10^3^; one and six weeks, respectively after the end of the treatment, reaching BLI values similar to non-infected animals (Fig 3B; bottom panel P<0.05).

Animals were sacrificed at different times after the end of treatment (48h, 1 week and 6 weeks posttreatment) and the organs (spleen and liver) were photographed after injecting D-luciferin *ex vivo* (Fig 3C). There was a significant marked reduction in the weight of the spleen, which reached values similar to those of the uninfected animals at 6 weeks after the end of the treatment (Fig 3D P<0.001). *Ex vivo* bioluminescent values recorded from treated and untreated animals over the time were plotted (Fig 3E) confirming the BLI reduction seen *in vivo*. The BLI decrease was significant at all analysed times in both organs (P<0.001) with the exception of the liver at 48h posttreatment that was not significant and liver at 6wpi (P<0.01). Both organs showed logarithmic reduction of BLI from the end of the treatment to 6 weeks later. *Ex vivo* parasite burden was estimated using limiting dilution assay confirming parasite load reductions of 98%, 99,9%, and 99,9%, at 48h, 1 week and 6 weeks postinfection (Fig 3F).

## Discussion

The introduction of new medicines against VL from the initial concept to public release is a time-consuming and expensive process. Moreover, the clinical recurrences after treatment failure and the emergence of resistances are worsened by the shortage of new clinical entities and the long period needed to release a new medicine [33]. To bridge the gap between early drug identification and *in vivo* preclinical studies, new bioimaging tools have recently been introduced to accelerate the drug discovery process while drastically reducing the number of animals used.

To develop robust preclinical *in vivo* platforms, several aspects related to the genetic modifications introduced in the pathogen and the suitability of the animal model should be addressed before their validation with a proof of concept [34, 35]. In such a way, we present here the generation of the strain *PpyRE9h+L*. *infantum* and its utility to quantify the parasite load *in vivo* in infected mice in real time. As the virulence of the modified strain can be lost after genetic manipulation and passage in culture, as soon as the correct integration of the construct was confirmed, the selected clone was passed through mice to recover its infectivity [36, 37, 38].

Once D-luciferin was administered, the light detected by CCD camera and transformed to pseudocolor images, enabled parasite traceability in the whole body and the estimation of parasite burden in a murine model of chronic VL, reducing the number of animals to be analysed in longitudinal studies. During *in vivo* infections amastigotes enter into a semi-quiescent physiological stage in which major energetic processes are specifically repressed [39], explaining the differences in light emission between metacyclic promastigotes and freshly isolated amastigotes. However, our results show that parasites emitted light enough to provide accurate and rapid radiance that allow the appraisal up to 21 wpi. In this study, light could be detected in Balb/c mice in the liver during the acute phase of infection and later in the spleen and bone marrow during the chronic phase, allowing a continuous and long-term follow-up of the infection. Under these conditions, light detected *in vivo*–that corresponded to ROIs drawn around liver and spleen—correlated well with parasite burden calculated from LDA, which it would allow to estimate the parasite burden without the sacrifice of animals.

The location of parasites (peripheral or deeper tissues) within the mammalian host has been pointed as a key factor affecting the limit of parasite detection *in vivo* [40]. The light emitted by freshly isolated amastigotes from splenic lesions in our system showed a detection threshold similar to the previously reported by other authors [22].

In experimental VL, the hamster is considered the best experimental model since it reproduces many clinicopathological features of the human disease and can be fatal in the absence of treatment [41]. High-dose murine models of VL develop hallmarks of progressive human, primate, and canine disease with loss of gp38 stromal cells [42], remodelling of splenic marginal zone region [43], altered migration of DCs [42] and loss of follicular germinal centers [44]. For this reason, Balb/c mice have been proposed as an adequate model of chronic VL [45–46]. In addition, during chronic infections the persistence of pathogens yields a state of T cell dysfunction known as exhaustion that is characterized by the loss of effector functions, low recall response and suboptimal T cell proliferation [28]. In VL, this stage of T cell exhaustion is associated with disease progression in mouse, dogs and human infections [29–31]. For this reason and in order to have a murine BLI model of chronic VL, the inoculum size was increased to 5x10^8^ metacyclic parasites per mouse.

In previous studies we have used the same *L*. *infantum*/Balb/c model showing hallmarks of progressive infection [47]. *PpyRE9h+L*. *infantum* strain allowed a continuous monitoring of parasite load from the beginning of the infection up to animal’s sacrifice, detecting both acute and chronic infections. In view of these results, *PpyRE9h+L*. *infantum* constitutes an ideal tool for the appraisal of drug efficacy in *in vivo* preclinical models.

The assay was validated by the treatment with miltefosine, starting 14 weeks post-infection and extended for long-term appraisal (6 weeks after drug withdrawal). In rodents miltefosine is known to produce significant parasite burden reduction (90–99% depending on parasite strain) in liver and spleen, along with no-sterile cure (when the treatment is initiated at 7–21 days postinfection [48–50]. These no-sterile curative results were confirmed later in hamster models of chronic VL treated with high-dose miltefosine (20 mg/kg/10 days), started 40 days post infection, although it resulted in 100% survival measured 20 wpi [23]. In our study, both radiance and parasite burden values dropped immediately after the end of treatment and they remained decreasing during the long-term follow up, although sterile cure was never achieved. A possible disease recurrence due to incomplete parasite suppression was expected. However, and despite the long-term follow up, much beyond the half-life of miltefosine [51], no recurrence was seen.

Several studies with AmBisome and stibogluconate in mice have shown that the infection status influenced treatment outcome, so that treatments were less effective in the chronic infection model than in acute infection models [52, 53]. The changes that occur in liver and spleen structure and function during early and late stages on infection might be the cause [46]. The mouse model proposed in this work would provide accurate information about potential drugs and their efficacy on the later stages of infection when it has been described that the efficacy of several drugs might be more compromised.

In conclusion, the gold standard methods used to evaluate the efficacy of antileishmanial drugs based on LDA or microscopic examination are laborious and time-consuming, have intrinsic variability, require intensive use of animals and cannot be monitored in real time. However, novel *in vivo* bioimaging models based on bioluminescent *L*. *infantum* parasites are highly sensitive, easily traceable, and yet provide statistically valuable outcomes with the use of far fewer animals than traditional methods. This technology is reproducible; less expensive because it reduces the number of animals needed, it is barely distressing for animals and can be easily adapted to different experimental models being thereby suitable to accelerate drug development.
